# A Rare Case Report of Contrast Media-induced Sympathetic Crashing Acute Pulmonary Edema

**DOI:** 10.5811/cpcem.38444

**Published:** 2025-05-06

**Authors:** Clates P. Adams, Christian I Wade

**Affiliations:** * Madigan Army Medical Center, Department of Emergency Medicine, Tacoma, Washington; † Brooke Army Medical Center, The San Antonio Uniformed Service Health Education Consortium, Department of Emergency Medicine, San Antonio, Texas

**Keywords:** SCAPE, pulmonary edema, hypoxia, contrast induced, case report

## Abstract

**Introduction:**

Sympathetic crashing acute pulmonary edema (SCAPE), also known as flash pulmonary edema or hypertensive acute heart failure, is a critical condition characterized by a rapid escalation of sympathetic outflow, excessive afterload, and worsening heart failure. Although rare, contrast media-induced pulmonary edema is a severe adverse reaction, occurring in 0.001–0.008% of patients receiving intravenous contrast and accounting for 10–20% of lethal contrast reactions.

**Case Report:**

A 70-year-old male developed acute respiratory distress shortly after undergoing an outpatient, contrast-enhanced computed tomography. Despite treatment for suspected anaphylaxis, the patient’s condition continued to deteriorate until a diagnosis of SCAPE was ultimately recognized. Treatment with high-dose nitroglycerin, non-invasive positive pressure ventilation (NIPPV), and eventual intubation resulted in the patient’s full recovery.

**Conclusion:**

This report highlights the importance of recognizing SCAPE in patients presenting with sudden dyspnea after contrast administration and emphasizes the need for early intervention with NIPPV and vasodilators to reduce morbidity and mortality.

## INTRODUCTION

Sympathetic crashing acute pulmonary edema (SCAPE), also known as flash pulmonary edema or hypertensive acute heart failure, is a critical and rapidly progressing condition. It is characterized by an acute increase in sympathetic outflow, excessive afterload, and worsening heart failure. Although SCAPE is rare, contrast media-induced SCAPE occurs in only 0.001–0.008% of all cases of SCAPE but accounts for 10–20% of lethal contrast reactions. 1–3

Sympathetic acute crashing pulmonary edema is more frequently seen in patients with chronic left ventricular dysfunction, particularly those with coexisting hypertension and renal artery stenosis. Additionally, factors that increase sympathetic tone and catecholamine release can precipitate SCAPE, creating a vicious cycle of dyspnea, anxiety, and worsening of the patient’s presentation.^4^,^5^ Early recognition and appropriate management are crucial to improving patient outcomes in such cases.

## CASE REPORT

A 70-year-old male with a history of coronary artery disease status post two stents in the right coronary artery, atrial fibrillation, hypertension, obstructive sleep apnea, and eosinophilic esophagitis presented to the emergency department (ED) in acute respiratory failure. Approximately 20 minutes before his presentation to the ED, the patient had received intravenous (IV) contrast for a routine outpatient computed tomography (CT) hematuria protocol. While undergoing the study, he experienced sudden-onset nausea, tachypnea, tachycardia, and respiratory distress.

Upon arrival to the ED, his first set of vitals were as follows: systolic blood pressure (BP), 151 millimeters of mercury (mm Hg); heart rate, 112 beats per minute; oxygen saturation, 74% on room air; and respiration rate, 30 breaths per minute. Physical examination was significant for an acutely distressed, diaphoretic male with increased work of breathing, diffuse rales in all lung fields, and tachycardia without murmurs, gallops, or rubs. There was no evidence of lower extremity edema, and no evidence of urticaria. He was immediately placed on nasal cannula. Given the recency of the contrasted CT study and his clinical presentation, an anaphylactic reaction was initially suspected. He was treated with three successive doses of intramuscular epinephrine (0.3 milligram [mg] per dose), nebulized ipratropium-albuterol (3 milliliters [mL]), diphenhydramine (50 mg), famotidine (20 mg), magnesium (2 grams), and methylprednisolone (125 mg). Despite these interventions, the patient’s respiratory failure persisted, leading to the initiation of an epinephrine drip and the application of a non-rebreather mask, although without improvement to his oxygen saturation.

Due to the patient’s worsening condition, which at that point was characterized by severe hypertension (systolic BP>200 mm Hg, partially secondary to our aforementioned interventions of epinephrine), rhonchorous breath sounds, and lack of wheezing, lack of urticaria, absence of mucosal edema, and lack of response to epinephrine, SCAPE was suspected. An electrocardiogram (ECG) and chest radiograph (CXR) were obtained. The ECG showed no signs of ischemia, while the CXR revealed significant right-sided pulmonary edema (Image). The epinephrine drip was discontinued, and the patient was administered IV furosemide (20 mg), followed by a nitroglycerin drip (starting at 5 micrograms per minute [mcg/min]), and placed on continuous positive airway pressure (CPAP).

To improve CPAP tolerance, the patient received lorazepam (1 mg), which temporarily stabilized oxygen saturation to the low 90s. However, the patient eventually became increasingly somnolent, with oxygenation saturations dropping to the mid-80s to low 90s. A venous blood gas revealed acute hypercapnic acidosis (pH 7.15, reference range: 7.29–7.45) and partial pressure of carbon dioxide 67 mm Hg (30.0–68.0 mm Hg), prompting the decision to intubate the patient. After successful intubation, the patient was placed on a ventilator, administered propofol, and admitted to the intensive care unit. There, a point-of-care echocardiogram demonstrated a normal left ventricular ejection fraction (>60%), a small-to-moderate pericardial effusion, and mild tricuspid regurgitation. The patient was eventually discharged home seven days after presentation.

*CPC-EM Capsule*

What do we already know about this clinical entity?

*Sympathetic Crashing Acute Pulmonary Edema (SCAPE) is a critical condition seen in emergency departments, characterized by rapid escalation of sympathetic outflow, excessive afterload, and worsening heart failure that requires prompt recognition and treatment*.

What makes this presentation of disease reportable?

*This presentation is reportable given the use of contrast-media, in which this presentation occurs in 0.001 - 0.008% of all patients receiving intravenous contrast, but accounts for 10*–*20% of lethal contrast reactions*.

What is the major learning point?

*The major learning point from this condition is early recognition of SCAPE, and prompt treatment with high-dose nitroglycerin and non-invasive positive pressure ventilation* (*NIPPV)*.

How might this improve emergency medicine practice?

*This case may improve emergency medicine practice to broaden the differential diagnosis of patients suffering from adverse contrast reactions, from the more common anaphylaxis*.

## DISCUSSION

Sympathetic acute crashing pulmonary edema is classified as a non-cardiogenic pulmonary edema and is thought to result from four primary mechanisms: catecholamine release, increased left atrial pressure leading to distension of small pulmonary capillaries, vascular endothelial cell damage causing interstitial edema, and increased microvascular permeability.^2^,^4^,^6^ The elevated catecholamine levels result in an increased heart rate, reduced diastolic time, and activation of the renin-angiotensin-aldosterone system, exacerbating diastolic stiffening and increasing diastolic and mean arterial pressures, thus contributing to pulmonary edema. Additionally, heightened sympathetic tone adversely affects pulmonary circulation by increasing permeability, provoking pulmonary capillary failure and causing splanchnic venoconstriction, further worsening dyspnea and sympathetic activation.^4^,^5^,^7^

The administration of contrast media triggers the release of inflammatory mediators and complement activation, leading to endothelial damage. This damage increases microvascular permeability, resulting in fluid accumulation in the lungs. The leakage of fluids from the circulation raises hemoglobin concentration and packed-cell volume, along with increased left atrial pressure. Furthermore, the complement system releases vasodilatory substances, increases vascular permeability and edema, promotes smooth muscle cell contraction, precipitates bronchospasm, and increases mucus secretion in the airways.^3^

Contrast-induced pulmonary edema is an exceedingly rare but highly lethal phenomenon, occurring in 0.001–0.008% of patients receiving IV contrast, with a mortality rate of 10–20%.^1^–^3^ Given the high frequency of contrast-enhanced studies performed in the ED, SCAPE should be a key differential diagnosis in patients presenting with any combination of sudden dyspnea, tachypnea, hypoxemia, rales, or hypertension. The treatment of SCAPE centers on oxygen administration via non-invasive positive pressure ventilation (NIPPV) or invasive ventilation with positive end-expiratory pressure and the use of high-dose vasodilators (such as nitroglycerin). Volume removal (diuresis or dialysis) may be indicated based on clinical judgement.^2^,^3^,^6^,^8^

Non-invasive positive pressure ventilation reduces the work of breathing, decreases preload and afterload, and has proven effective in preventing intubation.^8^ Studies indicate no significant difference in outcomes between bilevel positive airway pressure and continuous positive airway pressure.^8^ Vasodilators, such as nitroglycerin, relieve pulmonary congestion by reducing preload and afterload.^8^ The recommended initial dose is a bolus of 1,000–2,000 mcg over two minutes, followed by an infusion of 50–300 mcg/min, with rapid titration up to 800 mcg/min if needed, targeting a BP of less than 140 mm Hg.^5^,^8^ In cases of refractory hypertension, clevidipine (preferred) or nicardipine should be considered.^5^

## CONCLUSION

Sympathetic crashing acute pulmonary edema is a critical condition driven by a vicious cycle of increasing sympathetic outflow, excessive afterload, and worsening heart failure.^5^ When evaluating a patient for SCAPE, it is essential to differentiate it from angioedema and anaphylaxis, as the treatments differ significantly. Early recognition and prompt intervention, including the use of NIPPV and high-dose nitroglycerin, are crucial to improving patient outcomes and avoiding intubation and death.

## Figures and Tables

**Image:**
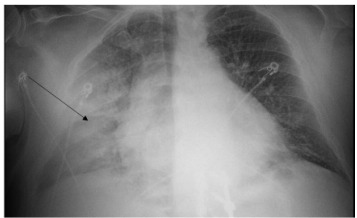
Initial emergency department chest radiograph with evidence of acute right-sided “flash” pulmonary edema (arrow).
